# Supplementary material for: Glucose uptake to guard cells via STP transporters provides carbon sources for stomatal opening and plant growth
Source: EMBO Rep. 2020 Jul 6;21(8):e49719. doi: 10.15252/embr.201949719 (PMC7403697; doi:10.15252/embr.201949719)
Supplement: Supplementary file 1 — Appendix [file EMBR-21-e49719-s001.pdf]

Appendix Flütsch *et al.*

Table of Contents:

Appendix Table S1: List of putative plasma membrane sugar transporter genes. Page 2

Appendix Table S2: Oligonucleotides used in this study. Page 3

Appendix Table S3: Leaf surface temperature, normalized leaf surface temperature and normalized differences in vegetation index NDVI. Page 4

Appendix Table S4: Chlorophyll fluorescence parameters. Page 5

Appendix Table S5: RGB parameters. Page 6

Appendix Figure S1: Gene Expression of *STPs* in *stp* mutant backgrounds. Page 7

Appendix References List Page 8

Gene	Name	AGI code
SUC1	Sucrose Transporter 1	AT1G71880
SUC2	Sucrose Transporter 2	AT1G22710
SUC3	Sucrose Transporter 3	AT2G02860
SUC5	Sucrose Transporter 5	AT1G71890
SUC8	Sucrose Transporter 8	AT2G14670
SUC9	Sucrose Transporter 9	AT5G06170
SWEET1	Sugars Will Eventually be Exported Transporters 1	AT1G21460
SWEET3	Sugars Will Eventually be Exported Transporters 3	AT5G53190
SWEET4	Sugars Will Eventually be Exported Transporters 4	AT3G28007
SWEET5	Sugars Will Eventually be Exported Transporters 5	AT5G62850
SWEET6	Sugars Will Eventually be Exported Transporters 6	AT1G66770
SWEET7	Sugars Will Eventually be Exported Transporters 7	AT4G10850
SWEET8	Sugars Will Eventually be Exported Transporters 8	AT5G40260
SWEET9	Sugars Will Eventually be Exported Transporters 9	AT2G39060
SWEET10	Sugars Will Eventually be Exported Transporters 10	AT5G50790
SWEET11	Sugars Will Eventually be Exported Transporters 11	AT3G48740
SWEET12	Sugars Will Eventually be Exported Transporters 12	AT5G23660
SWEET13	Sugars Will Eventually be Exported Transporters 13	AT5G50800
SWEET14	Sugars Will Eventually be Exported Transporters 14	AT4G25010
SWEET15	Sugars Will Eventually be Exported Transporters 15	AT5G13170
STP1	Sugar Transport Protein 1	AT1G11260
STP2	Sugar Transport Protein 2	AT1G07340
STP3	Sugar Transport Protein 3	AT5G61520
STP4	Sugar Transport Protein 4	AT3G19930
STP5	Sugar Transport Protein 5	AT1G34580
STP6	Sugar Transport Protein 6	AT3G05960
STP7	Sugar Transport Protein 7	AT4G02050
STP8	Sugar Transport Protein 8	AT5G26250
STP9	Sugar Transport Protein 9	AT1G50310
STP10	Sugar Transport Protein 10	AT3G19940
STP11	Sugar Transport Protein 11	AT5G23270
STP12	Sugar Transport Protein 12	AT4G21480
STP13	Sugar Transport Protein 13	AT5G26340
STP14	Sugar Transport Protein 14	AT1G77210
PMT1	Polyol/Monosaccharide Transporter 1	AT2G16120
PMT2	Polyol/Monosaccharide Transporter 2	AT2G16130
PMT3	Polyol/Monosaccharide Transporter 3	AT2G18480
PMT4	Polyol/Monosaccharide Transporter 4	AT2G20780
PMT5	Polyol/Monosaccharide Transporter 5	AT3G18830
PMT6	Polyol/Monosaccharide Transporter 6	AT4G36670

Appendix Table S1. List of (putative) plasma membrane sugar transporter genes in *Arabidopsis*.

Gene	AGI code	Forward primer	Reverse primer	PCR efficiency	Source
			**qPCR PRIMERS**		
ACT2	AT3G18780	CGTACAACCGGTATTGTGCT	GTAATCAGTAAGGTCACGTCCA	2.18	This study
BAM3	AT4G17090	TGATTCTGTGCCTGTCCT	GAATTTCCGCAATAACTCCTC	2.07	Ref [1]
KAT1	AT5G46240	AGCATGGGATGGGAAGAGTGGAG	AGAGCAGTGTCGGAAGTCGGAT	1.88	Ref [2]
MYB60	AT1G08810	CATGAAGATGGTGATCATGAGG	TTCCATTTGACCCCCAGTAG	1.98	Ref [3]
STP1	AT1G11260	GCTAATCAGGCTGTGCCACT	CAACCGCCTTTGATCTTGGC	2.09	This study
STP4	AT3G19930	TGCTTGTCTCTCAGATCGCTA	GCCACGATTAGATTGGCATCAC	2.01	This study
STP13	AT5G26340	TCAAGCCGTTCCGTTGTTCT	CCTTTAATCTTGGCGGTCCCA	1.96	This study
SUC1	AT1G71880	CATCCATATTCTCAAGCTGCTC	GCTAATACTCCATAATCGCC	2.10	Ref[4]
SUC3	AT2G02860	CAAGAACCGCAGCCGTAATC	CTTGACCGCCACCGGAAT	1.85	Ref[5]
		**GENOTYPING PRIMERS for amplification of gene-specific band**			
STP1	AT1G11260	ACACTCCCAATTCAATGATCG	TCATATGCATAATTTCTTCATGTGG		SIGnAL
STP4	AT3G19930	AAATTTTGTCCCCATTCCATG	AAAGCACCGATGAAACATGTC		SIGnAL
STP13	AT5G26340	CACGACTAGAATTGCGAAACC	AAAATTTTCTCCGGCATTGAC		SIGnAL
SUC1	AT1G71880	AGAGAGAGCTGTAAGGCCCAC	TACCATCTTTGGGACTGTTCG		SIGnAL
SUC3	AT2G02860	TCTATCACCTTCACAATCACACAC	ACCAGATTCGTTTTGACGATG		SIGnAL
		**GENOTYPING PRIMERS for amplification of T-DNA-specific band**			
STP1-1	AT1G11260	ATTTTGCCGATTTCGGAAC	TCATATGCATAATTTCTTCATGTGG		SIGnAL
STP4-1	AT3G19930	ATTTTGCCGATTTCGGAAC	AAAGCACCGATGAAACATGTC		SIGnAL
STP1-2	AT1G11260	TCATGATGACACACAAGGCTC	CCGATTGTAATTGAGAGCTGG		SiGnaL
STP4-2	AT3G19930	ATGTGAATGAAAAGGCTCACG	AAAGCACCGATGAAACATGTC		SiGnaL
STP13	AT5G26340	ATTTTGCCGATTTCGGAAC	AAAATTTTCTCCGGCATTGAC		SIGnAL

Appendix Table S2. Oligonucleotides used in this study.

Parameter	WT		*stp1-1*		*stp4-1*		*stp13*		*stp1stp4*		*stp1stp13*		*stp4stp13*	
	day 0	day 7	day 0	day 7	day 0	day 7	day 0	day 7	day 0	day 7	day 0	day 7	day 0	day 7
**Temp (°C)**	22.498 ± 0.078	22.409 ± 0.152	22.663 ± 0.098	22.500 ± 0.167	22.616 ± 0.082	22.606 ± 0.138	22.537 ± 0.079	22.443 ± 0.170	22.815 ± 0.102	22.652 ± 0.156	22.615 ± 0.068	22.457 ± 0.155	22.578 ± 0.066	22.510 ± 0.140
**Temp norm**	0.994 ± 0.001	0.996 ± 0.001	1.001 ± 0.001	1.000 ± 0.001	0.998 ± 0.002	0.999 ± 0.002	0.997 ± 0.001	0.998 ± 0.001	1.011 ± 0.002 *	1.008 ± 0.001 *	1.001 ± 0.001	0.999 ± 0.002	0.998 ± 0.001	1.001 ± 0.001
**NDVI**	0.756 ± 0.002	0.771 ± 0.003	0.754 ± 0.002	0.767 ± 0.002	0.746 ± 0.002	0.771 ± 0.002	0.747 ± 0.001	0.770 ± 0.003	0.739 ± 0.002	0.752 ± 0.004	0.759 ± 0.002	0.777 ± 0.003	0.754 ± 0.002	0.776 ± 0.002

Appendix Table S3. Leaf surface temperature (Temp), normalized leaf surface temperature (Temp norm) and normalized difference vegetation index (NDVI).
Parameters were calculated at the beginning (day 0, 3-week-old plants) and at the end (day 7, 4-week-old plants) of the phenotyping period in WT, *stp1-1, stp4-1, stp13, stp1stp4, stp1stp13* and *stp4stp13* plants. Data shown are means ± SEM; n = 10. Units are indicated next to each parameter. If absent, dimensionless. Within the same row and for the specified day, asterisk (*) indicates significant statistical difference between WT and *stp* plants for $P < 0.05$ determined by one-way ANOVA with *post hoc* Tukey's test. If absent, no statistical differences between the genotypes.

ChlF parameter	WT		*stp1-1*		*stp4-1*		*stp13*		*stp1stp4*		*stp1stp13*		*stp4stp13*	
	day 0	day 7	day0	day 7	day 0	day 7	day 0	day 7	day 0	day 7	day 0	day 7	day 0	day 7
F_0	99.82 ± 0.75	119.22 ± 1.36	93.55 ± 2.16	118.04 ± 1.51	94.08 ± 1.39	116.76 ± 1.10	98.79 ± 2.45	119.50 ± 1.45	94.74 ± 3.03	116.80 ± 2.20	99.69 ± 1.78	121.01 ± 1.52	101.48 ± 2.28	120.25 ± 1.58
F_m	582.28 ± 7.99	702.37 ± 15.77	546.06 ± 17.12	694.43 ± 12.01	549.53 ± 9.52	696.23 ± 11.95	571.58 ± 13.36	704.46 ± 11.45	526.20 ± 18.40	658.67 ± 17.42	579.98 ± 12.48	719.13 ± 12.91	595.99 ± 15.16	718.31 ± 13.17
F_0'	76.83 ± 0.62	92.80 ± 0.74	71.36 ± 1.51	91.20 ± 1.14	71.76 ± 1.23	89.65 ± 0.63	75.24 ± 2.06	92.97 ± 1.28	70.60 ± 2.13	89.11 ± 1.49	76.82 ± 1.39	94.28 ± 1.36	77.18 ± 1.59	93.32 ± 1.08
F_m'	214.12 ± 3.05	264.53 ± 2.42	195.71 ± 4.35	256.40 ± 3.77	197.21 ± 4.37	250.61 ± 2.49	205.41 ± 6.40	264.77 ± 4.94	183.20 ± 5.26 *	241.34 ± 3.81 *	214.81 ± 4.91	270.21 ± 5.62	211.76 ± 4.93	266.04 ± 3.87
F_q'	76.26 ± 2.32	95.64 ± 1.74	67.43 ± 1.93	91.12 ± 2.43	68.84 ± 2.55	89.01 ± 2.24	70.62 ± 3.30	95.67 ± 3.32	55.15 ± 1.83 *	77.78 ± 1.87 *	76.68 ± 3.15	98.91 ± 3.46	74.04 ± 3.17	97.75 ± 2.67
F_t'	137.87 ± 1.09	168.89 ± 1.33	128.28 ± 3.05	165.28 ± 1.92	128.37 ± 2.17	161.60 ± 1.23	134.79 ± 3.48	169.10 ± 2.09	128.05 ± 3.95	163.56 ± 2.42	138.14 ± 2.43	171.31 ± 2.43	137.72 ± 2.77	168.29 ± 1.97
F_v	482.45 ± 7.58	583.15 ± 14.55	452.51 ± 15.12	576.39 ± 10.75	455.44 ± 8.46	579.47 ± 10.97	472.78 ± 11.32	584.95 ± 10.63	431.45 ± 15.58	541.86 ± 15.48	480.28 ± 11.08	598.13 ± 11.62	494.51 ± 13.03	598.06 ± 11.90
F_v'	137.3 ± 2.58	171.73 ± 2.03	124.37 ± 2.96	165.20 ± 2.81	125.46 ± 3.22	160.96 ± 2.12	130.17 ± 4.43	171.80 ± 3.83	112.57 ± 3.22 *	152.23 ± 2.45 *	137.99 ± 3.73	175.93 ± 4.34	134.58 ± 3.68	172.72 ± 3.14
F_v/F_m	0.829 ± 0.002	0.829 ± 0.002	0.829 ± 0.002	0.829 ± 0.002	0.829 ± 0.002	0.832 ± 0.002	0.828 ± 0.002	0.830 ± 0.002	0.819 ± 0.002 *	0.821 ± 0.002 *	0.829 ± 0.002	0.831 ± 0.002	0.830 ± 0.002	0.832 ± 0.002
F_v'/F_m'	0.642 ± 0.003	0.650 ± 0.003	0.637 ± 0.003	0.645 ± 0.002	0.637 ± 0.003	0.643 ± 0.003	0.635 ± 0.003	0.650 ± 0.003	0.613 ± 0.003 *	0.631 ± 0.002 *	0.643 ± 0.004	0.652 ± 0.003	0.636 ± 0.005	0.650 ± 0.003
Φ_{PSII}	0.355 ± 0.006	0.361 ± 0.004	0.345 ± 0.006	0.356 ± 0.005	0.349 ± 0.006	0.355 ± 0.006	0.344 ± 0.007	0.361 ± 0.006	0.301 ± 0.006 *	0.321 ± 0.004 *	0.355 ± 0.007	0.366 ± 0.006	0.348 ± 0.009	0.367 ± 0.006
qL	0.993 ± 0.013	1.010 ± 0.008	0.975 ± 0.012	0.998 ± 0.010	0.977 ± 0.012	0.993 ± 0.011	0.966 ± 0.013	1.010 ± 0.012	0.869 ± 0.015 *	0.930 ± 0.009 *	0.989 ± 0.015	1.019 ± 0.011	0.972 ± 0.018	1.018 ± 0.012
qP	0.553 ± 0.007	0.555 ± 0.004	0.541 ± 0.008	0.551 ± 0.006	0.547 ± 0.008	0.552 ± 0.007	0.541 ± 0.008	0.556 ± 0.007	0.490 ± 0.009 *	0.509 ± 0.006 *	0.552 ± 0.009	0.561 ± 0.006	0.547 ± 0.010	0.565 ± 0.006
NPQ	1.734 ± 0.070	1.650 ± 0.066	1.801 ± 0.074	1.700 ± 0.055	1.802 ± 0.062	1.772 ± 0.057	1.802 ± 0.075	1.658 ± 0.068	1.903 ± 0.067	1.731 ± 0.049	1.717 ± 0.080	1.655 ± 0.056	1.826 ± 0.079	1.697 ± 0.066
qN	0.723 ± 0.010	0.710 ± 0.010	0.732 ± 0.010	0.718 ± 0.008	0.732 ± 0.008	0.727 ± 0.008	0.732 ± 0.010	0.710 ± 0.010	0.749 ± 0.008	0.723 ± 0.006	0.719 ± 0.011	0.711 ± 0.008	0.734 ± 0.011	0.716 ± 0.010
ETR	85.71 ± 1.43	87.06 ± 0.98	83.15 ± 1.51	85.89 ± 1.29	84.17 ± 1.46	85.62 ± 1.44	82.89 ± 1.68	87.14 ± 1.55	72.55 ± 1.56 *	77.55 ± 1.05 *	85.713 ± 1.790	88.238 ± 1.355	84.030 ± 2.149	88.565 ± 1.398

Appendix Table S4. Chlorophyll fluorescence (ChlF) parameters.

Parameters were measured at the beginning (day 0, 3-week-old plants) and at the end (day 7, 4-week-old plants) of the phenotyping period in WT, *stp1-1, stp4-1, stp13, stp1stp4, stp1stp13* and *stp4stp13* plants. Data shown are means ± SEM; n = 10. Apostrophe (’) indicates that the parameter was recorded in the light-adapted state at a photosynthetically active radiation (PAR) of 440 µmol m^{-2} s^{-1}. Within the same row and for the specified day, asterisk (*) indicates significant statistical difference between WT and *stp* plants for $P < 0.05$ determined by one-way ANOVA with *post hoc* Tukey’s test. If absent, no statistical differences between the genotypes.

RGB parameter	*WT* day 0	*WT* day 7	*stp1-1* day 0	*stp1-1* day 7	*stp4-1* day 0	*stp4-1* day 7	*stp13* day 0	*stp13* day 7	*stp1stp4* day 0	*stp1stp4* day 7	*stp1stp13* day 0	*stp1stp13* day 7	*stp4stp13* day 0	*stp4stp13* day 7
**Area (mm^2)**	327.45 ± 13.15^{c}	1419.38 ± 58.18^{c}	222.91 ± 6.13^{b}	1066.21 ± 35.03^{b}	239.02 ± 7.13^{b}	1062.40 ± 36.7^{b}	238.50 ± 5.77^{b}	1172.44 ± 24.71^{b}	151.76 ± 3.27^{a}	528.12 ± 17.52^{a}	313.76 ± 10.04^{c}	1430.91 ± 41.35^{c}	315.21 ± 8.52^{c}	1420.46 ± 32.97^{c}
**Growth rate (day^{-1})**	0.211 ± 0.003^{b}		0.222 ± 0.004^{bc}		0.212 ± 0.005^{b}		0.227 ± 0.002^{c}		0.175 ± 0.005^{a}		0.216 ± 0.003^{bc}		0.215 ±0.002^{bc}	
**Compactness**	0.52 ± 0.01	0.59 ± 0.01	0.54 ± 0.01	0.56 ± 0.01	0.51 ± 0.01	0.57 ± 0.01	0.52 ± 0.01	0.54 ± 0.004	0.50 ± 0.004	0.57 ± 0.02	0.51 ± 0.01	0.54 ± 0.01	0.50 ± 0.007	0.55 ± 0.01
**Eccentricity**	0.22 ± 0.02	0.19 ± 0.01	0.225 ± 0.019	0.183 ± 0.01	0.20 ± 0.01	0.20 ± 0.01	0.25 ± 0.01	0.20 ± 0.01	0.26 ± 0.01	0.22 ± 0.01	0.25 ± 0.01	0.18 ± 0.01	0.25 ± 0.01	0.19 ± 0.02
**Isotropy**	0.59 ± 0.03	0.62 ± 0.04	0.625 ± 0.027	0.595 ± 0.036	0.60 ± 0.03	0.58 ± 0.03	0.63 ± 0.03	0.54 ± 0.05	0.71 ± 0.03	0.61 ± 0.04	0.55 ± 0.03	0.52 ± 0.03	0.63 ± 0.02	0.52 ± 0.05
**Perimeter (mm)**	186.93 ± 8.11^{c}	431.90 ± 16.96^{cd}	158.10 ± 3.81^{b}	400.84 ± 7.62^{bc}	164.27 ± 4.36^{b}	377.06 ± 13.63^{b}	163.05 ± 2.87^{b}	415.63 ± 8.04^{bd}	130.70 ± 2.38^{a}	263.90 ± 10.34^{a}	191.77 ± 6.68^{c}	458.96 ± 10.21^{d}	186.18 ± 4.31^{c}	454.01 ± 6.60^{d}
**Rotational mass symmetry**	0.62 ± 0.02	0.47 ± 0.05	0.63 ± 0.03	0.35 ± 0.03	0.65 ± 0.02	0.47 ± 0.03	0.70 ± 0.03	0.45 ± 0.03	0.59 ± 0.04	0.54 ± 0.02	0.60 ± 0.05	0.44 ± 0.03	0.62 ± 0.03	0.47 ± 0.05
**Roundness**	0.12 ± 0.01	0.10 ± 0.01	0.11 ± 0.003	0.084 ± 0.003	0.11 ± 0.004	0.1 ± 0.005	0.11 ± 0.004	0.086 ± 0.003	0.11 ± 0.004	0.10 ± 0.006	0.11 ± 0.01	0.09 ± 0.004	0.12 ± 0.005	0.09 ± 0.003
**Roundness 2**	0.87 ± 0.01	0.88 ± 0.01	0.88 ± 0.01	0.88 ± 0.01	0.88 ± 0.002	0.88 ± 0.006	0.87 ± 0.005	0.88 ± 0.004	0.85 ± 0.003	0.87 ± 0.007	0.86 ± 0.006	0.88 ± 0.006	0.86 ± 0.006	0.88 ± 0.007
**Slenderness of leaves**	12.52 ± 0.50	27.17 ± 0.9^{bc}	11.54 ± 0.41	25.72 ± 1.40^{ab}	13.08 ± 0.88	24.86 ± 1.15^{ab}	13.40 ± 0.40	27.64 ± 0.92^{bc}	11.82 ± 0.39	22.27 ± 0.84^{a}	14.48 ± 0.54	28.60 ± 1.04^{bc}	13.88 ± 0.86	31.73 ± 1.33^{c}
**Hue 1 (% area pixels)**	5.22 ± 0.26^{ac}	2.88 ± 0.18	6.45 ± 0.25^{bce}	3.16 ± 0.12	6.61 ± 0.21^{de}	3.42 ± 0.08	6.28 ± 0.21^{bce}	3.15 ± 0.13	7.46 ± 0.52^{e}	3.55 ± 0.25	5.58 ± 0.31^{acd}	2.96 ± 0.19	5.18 ± 0.23^{ab}	3.21 ± 0.09
**Hue 2 (% area pixels)**	7.29 ± 0.29^{a}	3.97 ± 0.09	8.98 ± 0.44^{cde}	4.89 ± 0.17	8.98 ± 0.34^{bde}	4.80 ± 0.12	8.50 ± 0.28^{ae}	4.33 ± 0.14	10.77 ± 0.41^{f}	6.04 ± 0.22	7.78 ± 0.24^{abc}	4.24 ± 0.18	7.86 ± 0.17^{ad}	4.12 ± 0.15
**Hue 3 (% area pixels)**	27.43 ± 1.31^{ab}	19.14 ± 1.60^{ab}	27.81 ± 1.52^{ab}	18.75 ± 1.44^{ab}	27.02 ± 1.382^{ab}	22.86 ± 1.161^{b}	24.21 ± 1.57^{ab}	18.44 ± 1.97^{ab}	20.09 ± 2.18^{a}	16.56 ± 2.25^{a}	28.99 ± 1.48^{b}	19.96 ± 1.48^{ab}	25.38 ± 2.54^{ab}	24.37 ± 1.87^{b}
**Hue 4 (% area pixels)**	8.14 ± 0.62^{ab}	4.04 ± 0.37	8.37 ± 0.57^{ac}	4.21 ± 0.25	8.22 ± 0.33^{ac}	4.94 ± 0.58	6.87 ± 0.32^{a}	3.18 ± 0.09	10.52 ± 0.64^{bc}	6.41 ± 0.44	7.15 ± 0.42^{a}	3.51 ± 0.26	7.31 ± 0.31^{a}	3.52 ± 0.19
**Hue 5 (% area pixels)**	45.03 ± 2.01	57.76 ± 1.28	40.48 ± 2.00	58.69 ± 1.24	41.65 ± 1.96	56.34 ± 1.45	46.64 ± 2.06	58.32 ± 1.09	42.96 ± 3.21	54.81 ± 1.81	41.39 ± 2.24	59.00 ± 1.33	46.41 ± 3.88	54.19 ± 1.88

Appendix Table S5. RGB parameters.

Parameters were measured at the beginning (day 0, 3-week-old plants) and at the end (day 7) of the phenotyping period in WT, *stp1-1*, *stp4-1*, *stp13*, *stp1stp4*, *stp1stp13* and *stp4stp13* plants. Growth rates were determined using the exponential function. Data shown are means ± SEM; n = 10. Units are indicated next to each parameter. If absent, dimensionless. Within the same row and for the specified day, different letters indicate significant statistical differences for $P < 0.05$ determined by one-way ANOVA with *post hoc* Tukey's test. If absent, no statistical differences amongst the genotypes.

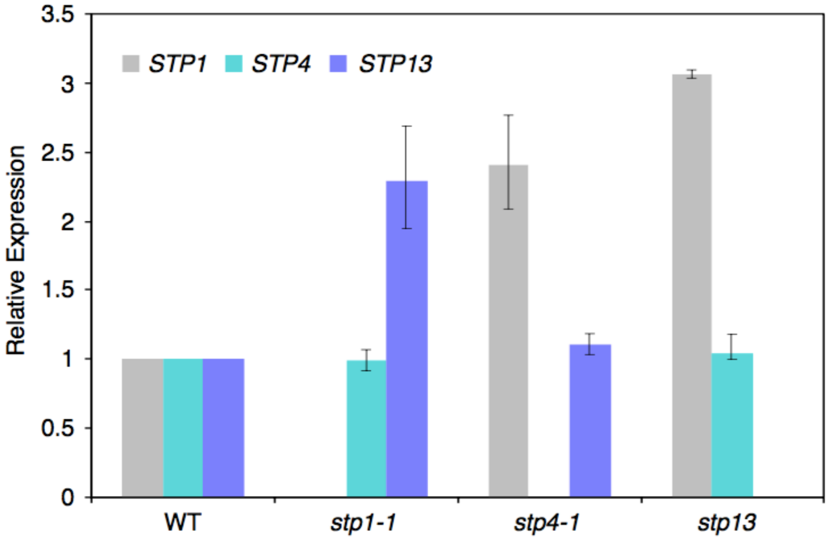

Appendix Figure S1. Gene Expression of *STPs* in *stp* mutant backgrounds.
STP1, *STP4* and *STP13* gene transcript levels in WT guard cell-enriched epidermal peels compared to *stp1-1, stp4-1* and *stp13* guard cell-enriched epidermal peels at the end of the night. *ACT2* was used as a housekeeping gene for normalization. Data for two independent experiments are shown; means ± fold change range n ≥ 4. For details about fold change and error calculations see Material and Methods section. Primer sequences and efficiencies are given in Appendix Table S2.

Reference List:

1. Yamada, K., Saijo, Y., Nakagami, H. & Takano, Y. Regulation of sugar transporter activity for antibacterial defense in *Arabidopsis*. *Science.* 354, 1427–1430 (2016).
2. Norholm, M. H. H., Nour-Eldin, H. H., Brodersen, P., Mundy, J. & Halkier, B. a. Expression of the *Arabidopsis* high-affinity hexose transporter STP13 correlates with programmed cell death. *FEBS Lett.* 580, 2381–7 (2006).
3. Schofield, R. a, Bi, Y.-M., Kant, S. & Rothstein, S. J. Over-expression of *STP13*, a hexose transporter, improves plant growth and nitrogen use in *Arabidopsis thaliana* seedlings. *Plant, Cell Environ.* 32, 271–85 (2009).
4. Bates, G.W. *et al.* A comparative study of *Arabidopsis thaliana* guard-cell transcriptome and its modulation by sucrose. *PLoS ONE* 7(11): e49641. doi:10.1371/journal.pone.0049641 (2012).
5. Dubey, N.K. *et al.* Expression Pattern of Sucrose Transporters in *Arabidopsis thaliana* during Aphid (*Myzus persicae*) infestation. *Amer. Jour. of Plant Sci.* 4, 47-51 (2013).
