# Supplementary material for: Glucose uptake to guard cells via STP transporters provides carbon sources for stomatal opening and plant growth
Source: EMBO Rep. 2020 Jul 6;21(8):e49719. doi: 10.15252/embr.201949719 (PMC7403697; doi:10.15252/embr.201949719)
Supplement: Supplementary file 2 — Expanded View Figures PDF [file EMBR-21-e49719-s002.pdf]

Expanded View Figures

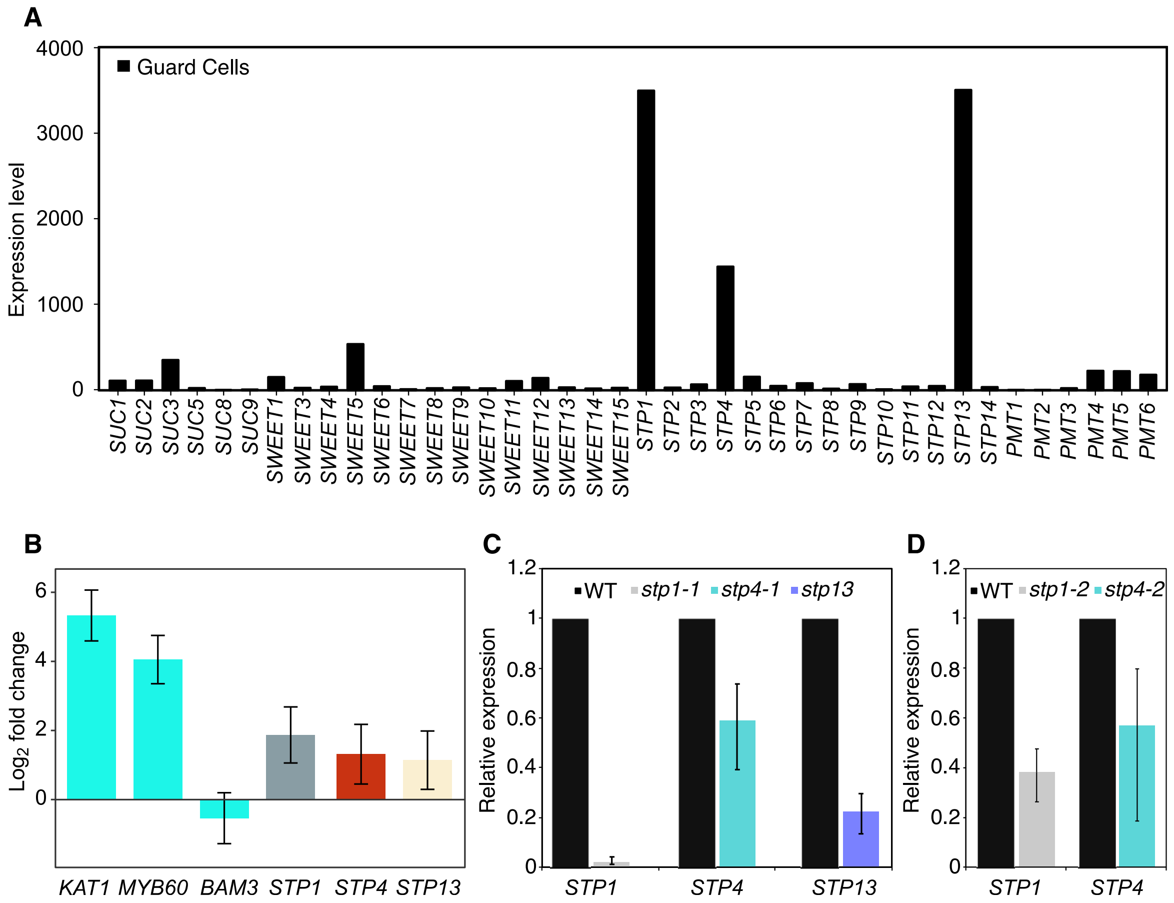

Figure EV1. ***STP1*, *STP4* and *STP13* are highly and preferentially expressed in guard cells.**

A *In silico* analysis of plasma membrane sugar transporter gene expression levels in *Arabidopsis* guard cells. *Arabidopsis* eFP browser (http://bar.utoronto.ca/efp2/Arabidopsis/Arabidopsis_eFPBrowser2.html); *Arabidopsis* guard cell protoplasts (Yang *et al*, 2008).

B *STP1*, *STP4*, and *STP13* gene transcript levels in WT guard cell-enriched epidermal peels compared to WT rosette leaves at the end of the night. *KAT1* and *MYB60* were used as guard cell-specific markers, whereas *BAM3* was used as leaf-specific marker. Data for two independent experiments are shown; means ± fold change range $n \geq 6$.

C *STP1*, *STP4*, and *STP13* gene transcript levels in WT rosette leaves compared to *stp1-1*, *stp4-1*, and *stp13* rosette leaves at the end of the night. Data for two independent experiments are shown; means ± fold change range $n \geq 6$.

D *STP1* and *STP4* gene transcript levels in WT rosette leaves compared to *stp1-2* and *stp4-2* rosette leaves at the end of the night. Data for two independent experiments are shown; means ± fold change range $n \geq 5$.

Data information: (B, C, and D) *ACT2* was used as a housekeeping gene for normalization. For details about fold change and error calculations, see Materials and Methods section. Primer sequences and efficiencies are given in Appendix Table S2.

 © 2020 The Authors

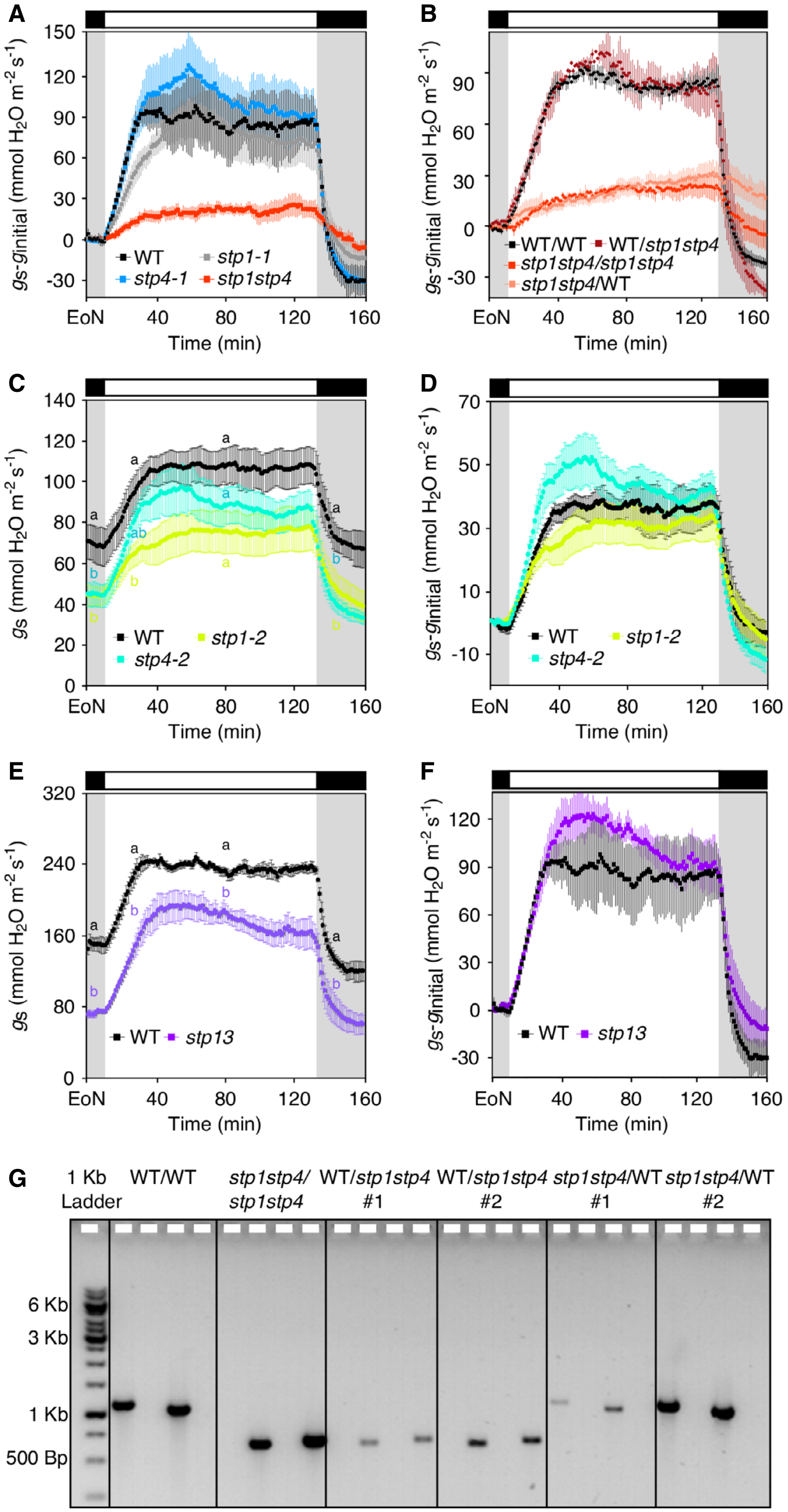

Figure EV2. Stomatal function in *stp13* plants and genetic identity of WT-*stp* grafted plants.

A Normalized whole-plant recordings of changes in stomatal conductance (g_s-$g_{initial}$) from WT, *stp1-1*, *stp4-1*, and *stp1stp4* plants. Data shown are means ± SEM; $n \geq 3$ per genotype.

B Normalized whole-plant recordings of changes in stomatal conductance (g_s-$g_{initial}$) from self-grafted donor lines (WT/WT, *stp1stp4/stp1stp4*) and reciprocal grafting of shoot/root (WT/*stp1stp4*, *stp1stp4*/WT) plants. Data shown are means ± SEM; $n \geq 3$ per genotype.

C Whole-plant recordings of changes in stomatal conductance (g_s) from WT, *stp1-2* and *stp4-2* plants. Data shown are means ± SEM; $n = 4$ per genotype.

D Normalized whole-plant recordings of changes in stomatal conductance (g_s) from WT, *stp1-2* and *stp4-2* plants. Data shown are means ± SEM; $n = 4$ per genotype.

E Whole-plant recordings of changes in stomatal conductance (g_s) from WT and *stp13* plants. Data shown are means ± SEM; $n = 3$ per genotype.

F Normalized whole-plant recordings of changes in stomatal conductance (g_s) from WT and *stp13* plants. Data shown are means ± SEM; $n = 3$ per genotype.

G Representative molecular genotyping of roots from reciprocal grafted WT/*stp1stp4* and *stp1stp4*/WT (shoot/root) plants. Roots from WT/WT and *stp1stp4/stp1stp4* were used as a control. Genomic DNA extracted from roots was amplified using the genotyping primers listed in Appendix Table S2. For each grafted plant, PCR products were loaded according to the following order: *STP1* gene-specific band, *STP1* T-DNA-specific band, *STP4* gene-specific band, *STP4* T-DNA-specific band.

Data information: (A - F) Plants have been illuminated with 150 µmol/m^2/s white light after the end of the night (EoN) under ambient-air CO_2 concentrations. (A, B, D, and F) g_s values were normalized to values at the end of the night (EoN; 0 = $g_{initial}$). (C and E) Different letters indicate significant statistical differences among genotypes for the given time point for $P < 0.05$ determined by one-way ANOVA with post hoc Tukey's test. (E and F) WT recordings are taken from Fig 1C.

© 2020 The Authors

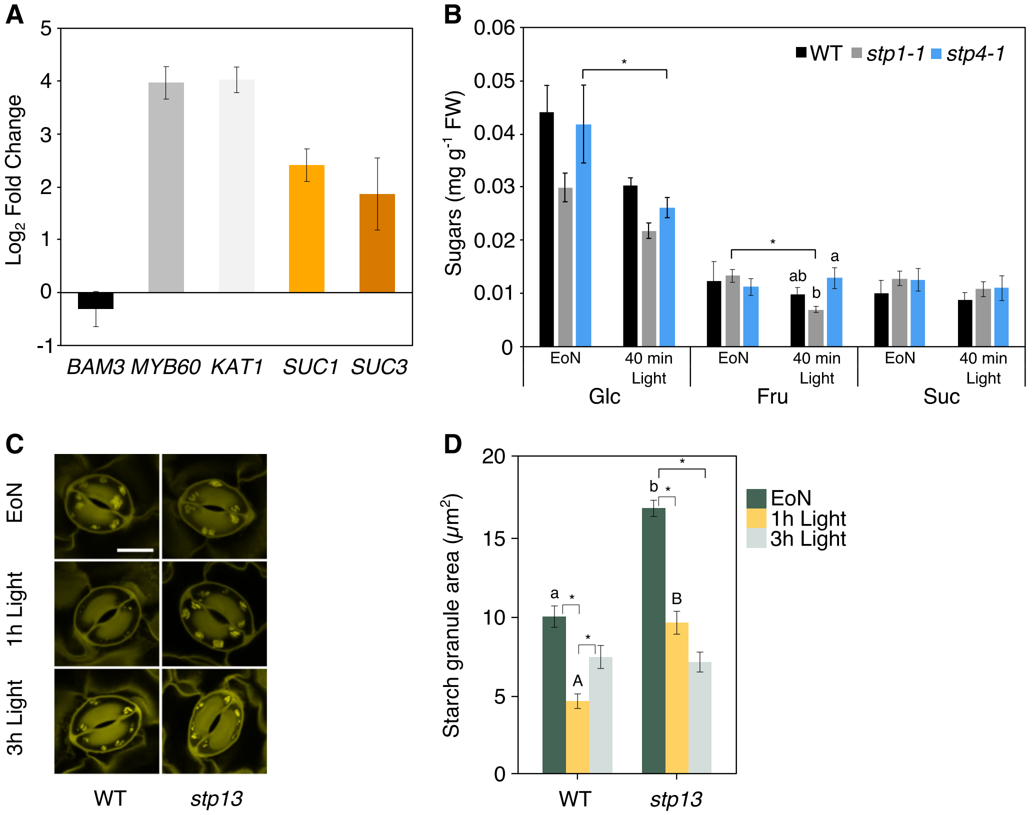

Figure EV3. Guard cell metabolites and gene expression in *stp* mutants.

A *SUC1* and *SUC3* gene transcript levels in WT guard cell-enriched epidermal peels compared to WT rosette leaves at the end of the night. *KAT1* and *MYB60* were used as guard cell-specific markers, whereas *BAM3* was used as leaf-specific marker. Data for two independent experiments are shown; means ± fold change range n ≥ 5.

B Content of soluble sugars in guard cell-enriched epidermal peels of WT, *stp1-1*, and *stp4-1* plants at the end of the night (EoN) and after 40 min of illumination with white light at 150 µmol/m^2/s following the EoN. Data shown are means ± SEM; *n* = 3 for WT and *n* ≥ 5 for the mutants per time point.

C Representative confocal laser microscopy images of propidium iodide-stained guard cell starch granules of intact leaves of WT and *stp13* plants. Scale bar, 10 µm.

D Starch dynamics in guard cells of intact leaves of WT and *stp13* plants at the end of the night (EoN) and after 1 and 3 h of illumination with 150 µmol/m^2/s of white light. Data for three independent experiments are shown; means ± SEM; *n* = 120 individual guard cells per genotype and time point.

Data information: (A) *ACT2* was used as a housekeeping gene for normalization. For details about fold change and error calculations, see Materials and Methods section. Primer sequences and efficiencies are given in Appendix Table S2. (B, D) Different letters indicate significant statistical differences among genotypes for the given time point. Asterisk (*) indicates significant statistical differences among time points for the given genotype for $P < 0.05$ determined by one-way ANOVA with post hoc Tukey's test. (C, D) WT data are taken from Fig 2B and C.

 © 2020 The Authors

Figure EV4. Carbohydrate metabolism in the leaves of WT and *stp* mutant plants.

A Whole-plant estimations of the internal CO_2 concentration (C_i) from WT, *stp1-1*, *stp4-1*, and *stp1stp4* plants. Data shown are means ± SEM; $n \geq 3$ per genotype.

B Representative false color images of photosystem II (PSII) operating efficiency (ΦPSII) captured by chlorophyll fluorescence imaging from WT, *stp13*, *stp1stp13*, and *stp4stp13* plants. ΦPSII was measured at a photosynthetically active radiation (PAR) of 440 μmol/m^2/s.

C ΦPSII quantified over the phenotyping period. Data shown are means ± SEM; n = 10 per genotype and time point.

D Normalized whole-plant recordings of changes in CO_2 assimilation (A) from WT, *stp1-1*, *stp4-1*, and *stp1stp4* plants. Data shown are means ± SEM; $n \geq 3$ per genotype.

E Normalized whole-plant recordings of changes in CO_2 assimilation (A) from self-grafted donor lines (WT/WT, *stp1stp4*/*stp1stp4*) and reciprocal grafting of shoot/root (WT/*stp1stp4*, *stp1stp4*/WT) plants. Data shown are means ± SEM; n = 3 per genotype.

F Whole-plant recordings of changes in CO_2 assimilation (A) from WT, *stp1-2*, and *stp4-2* plants. Data shown are means ± SEM; n = 4 per genotype.

G Normalized whole-plant recordings of changes in CO_2 assimilation (A) from WT, *stp1-2*, and *stp4-2* plants. Data shown are means ± SEM; n = 4 per genotype.

H Whole-plant recordings of changes in CO_2 assimilation (A) from WT and *stp13* plants. Data shown are means ± SEM; n = 3 per genotype.

I Normalized whole-plant recordings of changes in CO_2 assimilation (A) from WT and *stp13* plants. Data shown are means ± SEM; n = 3 per genotype.

J Quantification of leaf starch. Data shown are means ± SEM; n = 8 per genotype and time point.

K Quantification of leaf glucose (Glc). Data shown are means ± SEM; n = 8 per genotype and time point.

L Quantification of leaf fructose (Fru). Data shown are means ± SEM; n = 8 per genotype and time point.

M Quantification of leaf sucrose (Suc). Data shown are means ± SEM; n = 8 per genotype and time point.

N Representative Red Green Blue (RGB) images of 3-(day 0) and 4-week-old (day 7) WT, *stp13*, *stp1stp13*, and *stp4stp13* plants, Scale bar, 10 μm.

O Projected rosette area over the phenotyping period. Data shown are means ± SEM; n = 10 per genotype and time point.

Data information: (A and D – I) Plants have been illuminated with 150 μmol/m^2/s white light after the end of the night (EoN) under ambient-air CO_2 concentrations. (D, E, G, and I) A values were normalized to values at the end of the night (EoN; 0 = $A_{initial}$). (F and H) Different letters indicate significant statistical differences among genotypes for the given time point for $P < 0.05$ determined by one-way ANOVA with post hoc Tukey's test. (H and I) WT recordings are taken from Fig 3C and D. (J–M) Metabolites are from entire rosettes of WT, *stp13*, *stp1stp13*, and *stp4stp13* plants at the end of the night (EoN) and end of the day (EoD) in a 12-h light/12-h dark cycle. FW, fresh weight. Asterisk (*) indicates significant statistical difference between time points for the given genotype. Different letters indicate significant statistical differences among genotypes for the given time point for $P < 0.05$ determined by one-way ANOVA with post hoc Tukey's test.

© 2020 The Authors

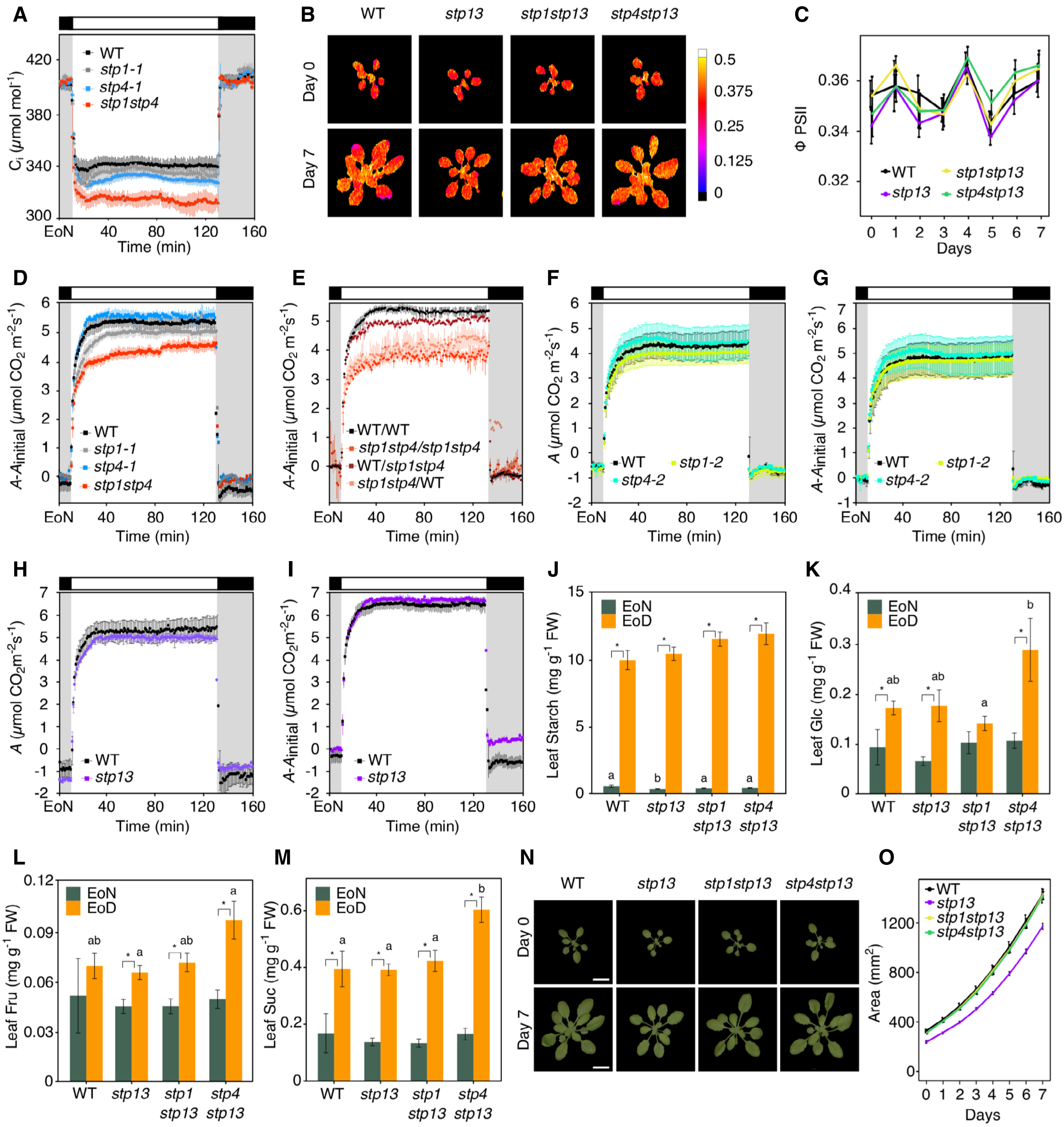

Figure EV4.

 © 2020 The Authors

Figure EV5. Variation in rosette colors and chlorophyll quantification in WT and *stp* plants.

A Representative false color images of normalized difference vegetation index (NDVI) captured by a visible-near-infrared (VNIR) hyperspectral camera in *stp* plants.
B NDVI measured over the phenotyping period. Data shown are means ± SEM; n = 10 per genotype and time point.
C–I Dynamic relative changes in greenness hue abundance over the phenotyping period in (C) WT; (D) *stp1-1*; (E) *stp4-1*; (F) *stp13*; (G) *stp1stp4*; (H) *stp1stp13*; (I) *stp4stp13*. The 5 most representative hues of green are shown in false color scale as percentage of the rosette area (pixel counts); n = 10.
J Chlorophyll *a*, *b* and *a+b* content in rosette leaves of WT and *stp* plants. Data for three independent experiments are shown; mean ± SEM; $n \geq 8$.

Data information: (B) Asterisk (*) indicates significant statistical difference between WT and *stp* plants for $P < 0.05$ determined by one-way analysis of variance (ANOVA) with *post hoc* Tukey's test. (J) FW, fresh weight.

© 2020 The Authors

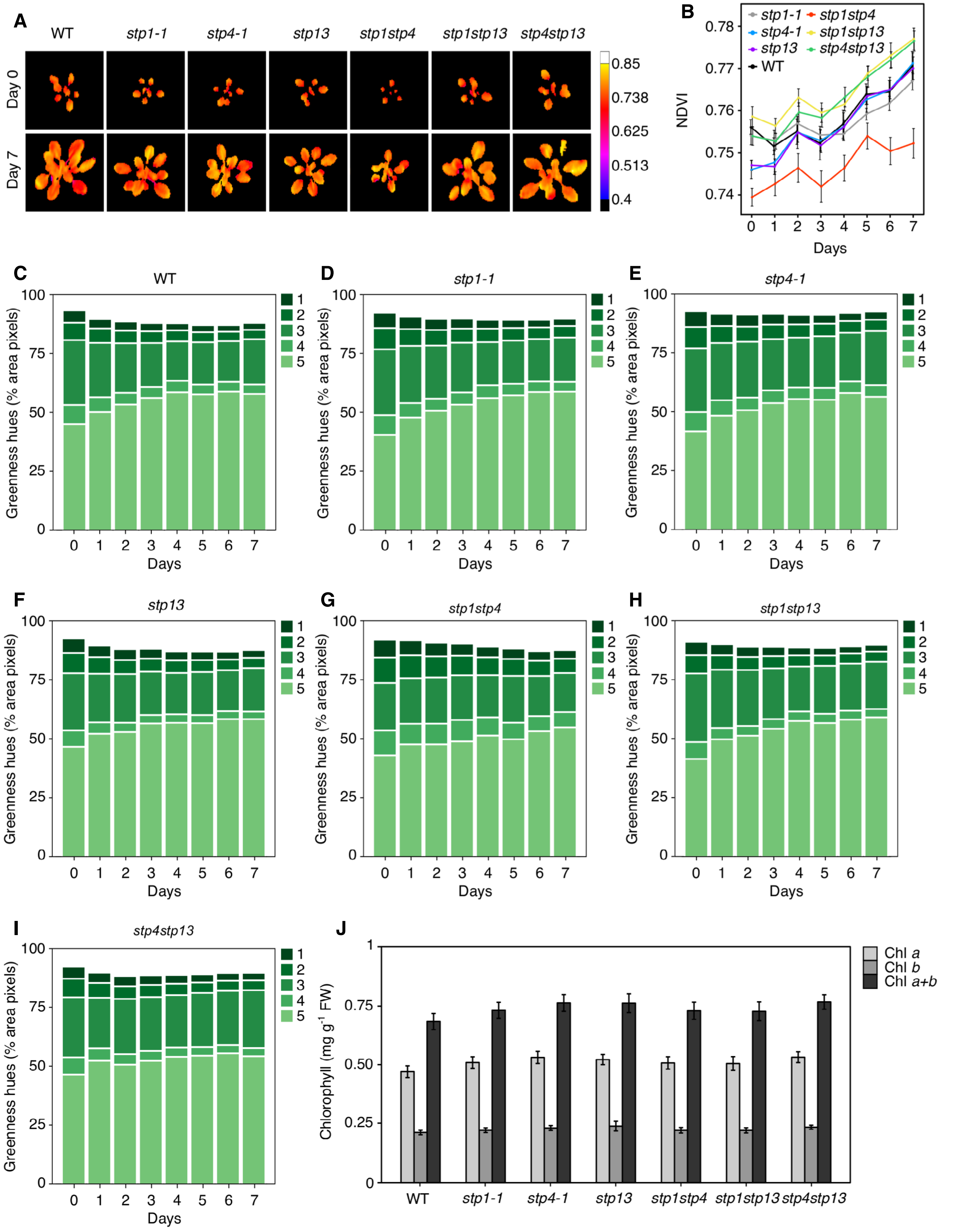

Figure EV5.

© 2020 The Authors
